# Effect of temperature variation on hormonal concentration at various gestation stages in black Bengal goat

**DOI:** 10.14202/vetworld.2015.1137-1142

**Published:** 2015-09-30

**Authors:** Binod Kumar, Ajay Kumar Ishwar, Pankaj Kumar Choudhary, Tanveer Akhatar

**Affiliations:** 1 Department of Veterinary Physiology, College of Veterinary Science &Animal Husbandry, Birsa Agricultural University, Ranchi, Jharkhand, India; 2 Department of Veterinary Physiology and Biochemistry, College of Veterinary Science & Animal Husbandry, Narendra Dev University of Agriculture and Technology, Kumarganj, Faizabad, Uttar Pradesh, India; 3 Department of Livestock Production and Management, College of Veterinary Science & Animal Husbandry, Birsa Agricultural University, Ranchi, Jharkhand, India

**Keywords:** black Bengal goat, estrus, hormonal profile, parturition, temperature variation

## Abstract

**Aim:**

The present study was conducted to evaluate the effect of risingtemperature on the metabolic as well as the reproductive performance of the black Bengal goat.

**Materials and Methods:**

A total 27 numbers of non-pregnant black Bengal goats of the same parity comprised the experimental animals. The selected goats were randomly assigned to 3 groups of 9 each, maintaining uniformity in body weight (average 14-18 kg). Goats in Group-I were kept between the temperature ranges of 35-40°C, in Group-II between 20°Cand 27°C, and Group-III were kept under loose housing system and serve as a control. Goats in all the groups were bred naturally. Blood was collected prior to feeding in the morning on the day 1 (estrus), 20, 45, 90, and 135, expected day of parturition and also 2 days after parturition from goats of all the three groups.

**Results:**

It was observed that the level of plasma estrogen decreased (p<0.05) up to day 45 of gestation, then after increased up to 135 days of gestation and was maximum on expected day of parturition which was significantly (p<0.05) higher than all the values. Plasma progesterone level increased from day 20 and was the highest on day 90 and then decreased significantly (p<0.05) on expected date of parturition. The luteinizing hormone value decreased significantly (p<0.05) on expected day of parturition and day 2 after parturition in all the groups. Follicle stimulating hormone concentration showed a significant (p<0.05) decrease from day 1 to 2 days after parturition in all the groups. The plasma triiodothyronine (T_3_) level did not vary between and within the treatment groups at any stage of the experiment. The plasma thyroxine (T_4_) level varied significantly (p<0.01) within and (p<0.05) between groups at all stages of reproduction. A significant (p<0.05) variation in plasma cortisol concentration in all the groups increased significantly until the day of parturition and dropped significantly (p<0.01) in 2 days after parturition in all the groups.

**Conclusion:**

The present experiment revealed that rise in temperature has no any deleterious effect on the metabolic as well as the reproductive hormonal concentrationat variousstages of gestation inblack Bengal goat.

## Introduction

Reproduction in farms animals is highly affected by environmental factors and when environmental conditions are favorable, reproductive activity expresses its full potential. Inadequate conditions may lead to a decrease in reproductive capacity, varying from sub-fertility to infertility. Inefficient reproduction may be caused by numerous factors which include environmental stressors such as temperature extremes, light intensity, and humidity. Reduced reproductive efficiency can occur as a result of environmental and managemental factors or stressors associated with animal housing and temperature extremes. Stimuli that challenge homeostasis like heat is commonly called stressors. Such stress can disrupt the physiology and productive performance of an animal [1]. These stressors cause deviation in the hormonal pattern and clinical manifestations [2]. Pregnancy or gestation is one of the important periods of any living organism. It depends mainly on the level of hormones and adequate nutrition [3].

In tropical and sub-tropical regions, the high ambient temperature is the major constraint on animal production [4]. Although goats are resistant to thermal stress at a greater extent, but they suffer from heat and cold stress beyond their comfort zone, which is environmental temperature 13-27°C for Indian goat [5]. Goat production in India makes a major contribution to the agrarian economy [6]. Here we choose among the several goat breeds of India, black Bengal goats [7] majorly distributed throughout eastern India and best in terms of meat and leather quality. Several biochemical markers are identified to assess the health status of goat exposed to stress [8]. Heat and humidity place a direct stress on these animals particularly at the time of grazing in the pasture and on the field conditions. The earth’s climate has warmed in the last century (0.74°C±0.18°C) with the 1990s and 2000s being the warmest on instrumental record (Intergovernmental Panel on Climate Change) [9]. Current climate models indicated an increase in temperature by 0.2°C per decade and predicted that the increase in global average surface temperature would be between 1.8°C and 4.0°C by 2100 [9].

The different phases of the reproductive cycle are regulated by sequential events and interaction between hypothalamic releasing hormones. Asound knowledge of reproductive functioning in terms of the interplay of hypothalamic, gonadotropic and gonadal hormones, with synergistic and antagonistic influence from other hormones and factors involved in regulation of various reproductive stages [10]. Many genetic, environmental and physiological factors affect the reproductive efficiency of afemale goat. Heat stress can lead to disruption in the reproductive process through two general mechanisms: Homeokinetic changes to regulate body temperature and failure of homeokinetic system to regulate reproduction. Considering the potential impact of temperature variation on black Bengal goat and scanty information regarding hormonal profile across the gestation period, a study was undertaken to report the hormones pregnant black Bengal at different environmental temperature.

## Materials and Methods

### Ethical approval

The experiment followed the guidelines of Institutional Animal Ethics Committee, and experimental plan followed the ethical guidelines on the proper care as well as the use of animal.

### Animals and treatment

The present study was conducted in the 27 cyclic, healthy black Bengal goats of similar age between 2 and 4 years and body weight (average 14-18 kg) maintained at Instructional Farm of Small Ruminant (IFSR), Ranchi Veterinary College, Birsa Agricultural University, Ranchi,India. They were divided randomly into three group (Group-I, Group-II, and Group-III) having 9 in each. Goats in Group-I were kept between the temperature range of 35°Cand 40°C with the help of electrical heater for 8 h per day during the period of experimentation. Goats in Group-II were kept under semi-intensive system temperature range between 20°Cand 27°C, the temperature was maintained by providing gunny bags on asbestos roof and floor of shed was provided with 1.5” fine sand for cooling effect. Animals of Group-III were kept under loose housing system and serve as a control. Animals were maintained on a standard ration [11] at the rate of 250g concentrate/animal/day with green fodder and water *ad libitum*. Blood (10ml) was collected by jugular venipuncture of each animal using disposable syringes and sterile needles 18 gauge×11/2”, prior to feeding in the morning on the day 1 (estrus), 20, 45, 90, and 135, expected day of parturition and also 2 days after parturition from goats of all the three groups. The collected blood samples were taken immediately to the laboratory in the ice box. Plasma was separated by centrifugation at 3000 rpm for 20 min and stored at −20°C±5°C until analysis.

### Observations

Estrus was detected by exposing the experimental animals to a teaser buck at 6 A.M., 12 A.M., and 6 P.M. The estrus lengths of the entire goat coming into estrus were recorded in hours. Goats in all the groups were bred naturally on the occurrence of estrus. Temperature humidity index (THI) was recorded fortnightly using the formula as described by Johnson [12]. T_3_ and T_4_ were estimated by radioimmunoassay method whereasestrogen, progesterone, follicle stimulating hormone (FSH), luteinizing hormone (LH), and cortisol were estimated by enzyme-linked immunosorbent assay technique at Nuclear Research Laboratory, Division of Physiology and Climatology, Indian Veterinary Research Institute, Izatnagar, Bareilly, Uttar Pradesh.

### Statistical analysis

The data were statistically analyzes by Analysis of Variance(ANOVA) test and significance was tested at 1% and 5% level as per the method described by Snedecor and Cochran [13].

## Results and Discussion

The average THI in different groups recorded at the different interval is present in Table-1. Marai and Haeeb [14] reported a high ambient temperature evokes a series of drastic changes in buffalo’s biological function which disturb the hormonal secretion as well as blood metabolites. Our findings are in agreement with finding of Marai and Haeeb [14] and Verma [15]. The average duration of estrus is present in Table-1. Our finding is in agreement with finding of Perera *et al*. [16] they reported the average duration of estrus between 19.00 and 32.00 h in the goat. The average plasma estrogen concentrations of day1 of estrus in different groups are presented in Table-2 (42.32±2.10, 51.47±3.03, 48.72±4.13 pg/ml). Our study revealed plasma estrogen concentration increased on expected days of parturition in all groups, and it was highly significant (p<0.01).

**Table-1:** Mean±SE of THI and estrus length of different housing system in different groups.

Parameter	Groups		

	Group-I (hot and humid)	Group-II (cold treatment)	Group-III (control)
THI	84.07±1.71	76.93±1.60	79.93±1.61
Estrus length (h)	28.63±3.44	26.51±1.51	32.01±2.49

SE=Standard error, THI=Temperature humidity index

**Table-2:** Mean±SE of plasma estrogen, plasma progesterone, LH, and FSH of black Bengal goats in different groups at different periods.

Parameters	Experimental group	Period of observations						

		Day 1 estrus	Day 20	Day 45	Day 90	Day 135	Expected day of parturition	2 days after parturition
Plasma estrogen (pg/ml)	Group-I	42.32±2.10^c^	9.865±1.64^a^	8.72±1.49^a^	15.82±3.56^ab^	26.22±2.52^ab^	258.45±8.88^e^	85.72±3.32^d^
	Group-II	51.47±3.03^c^	10.04±1.01^ab^	8.72±0.99^a^	15.70±2.33^ab^	23.02±1.77^b^	260.77±4.96^e^	89.15±2.34^d^
	Group-III	48.72±4.13^a^	10.87±1.35^a^	9.32±1.20^a^	14.74±2.98^ab^	24.82±2.43^b^	262.87±6.17^e^	86.64±1.17^d^
Plasma progesterone (ng/ml)	Group-I	0.20±0.01^a^	3.17±0.32^c^	3.56±0.21^cd^	4.13±0.48^d^	3.27±0.39^cd^	1.15±0.22^b^	0.60±0.16^ab^
	Group-II	0.17±0.02^a^	4.28±0.76^bc^	4.38±0.71^c^	5.12±0.61^d^	3.51±0.22^b^	1.23±0.22^a^	0.63±0.17^a^
	Group-III	0.19±0.02^a^	3.63±0.23^c^	4.43±0.70^cd^	5.29±0.62^d^	3.31±0.36^c^	1.47±0.38^b^	0.61±0.17^ab^
LH (ng/ml)	Group-I	0.82±0.05^cd^	0.56±0.01^b^	0.52±0.01^b^	0.30±0.01^a^	0.27±0.00^a^	0.74±0.03^c^	0.85±0.04^d^
	Group-II	0.84±0.05^cd^	0.55±0.01^b^	0.48±0.02^b^	0.30±0.01^a^	0.28±0.01^a^	0.80±0.03^c^	0.89±0.04^d^
	Group-III	0.87±0.05^d^	0.56±0.01^b^	0.49±0.02^b^	0.32±0.01^a^	0.29±0.01^a^	0.70±0.03^c^	0.82±0.05^d^
FSH (ng/ml)	Group-I	107.64±2.8^d^	53.34±1.15^b^	35.52±0.92^a^	72.86±0.85^e^	60.73±0.53^c^	37.39±0.07^a^	34.31±1.13^a^
	Group-II	112.11±4.34^d^	51.44±1.31^b^	34.33±1.02^a^	71.84±0.97	61.06±1.40^c^	37.50±1.17^a^	35.39±1.22^a^
	Group-III	113.11±4.66^d^	52.00±2.45^b^	33.87±1.03^a^	72.30±0.85^e^	61.12±0.73^c^	39.30±0.87^a^	37.23±0.77^a^

Mean values bearing different superscripts in a row differ significantly (p<0.05), FSH=Follicle stimulating hormone, LH=Luteinizing hormone, SE=Standard error

The plasma estrogen concentrations declined from day 20 to day 45 four prepartum followed by a continuous increase from day 90 to day 1 and then abrupt increase on the day of kidding. After kidding the levels declined on day 2 post-partum, this was maintained at the basal level up to day 20 post-partum. The prepartum increase and spurt in the level of estradiol-17β on the day of parturition is in general agreement with the findings in goats [17,18].

The average of total plasma progesterone concentration in different treatment groups recorded at the different interval is presented in Table-2. The level of plasma progesterone significantly increased (p<0.05) from day 20 to day 90 of gestation compare to the value on the day at estrus. Although, there was no significant decrease up to135 days but the values started declining thereafter, on the expected day of parturition and 2 days after parturition. The difference between mid and late gestation was significant (p<0.01). The difference between early and mid-pregnancy was not significant. The difference between early and late also between mid and late was significant (p<0.01). Our findings center the finding of Franandez *et al*.[19] in Spanish Ibex goats, Juarez *et al*. [20] and Gaafar *et al*.[21] in lactating goatand also as Verma [15] in ewes.

The average of total plasma LH concentration in different treatment groups recorded at the different interval is presented in Table-2. Plasma LH concentration did not vary significantly among different group of animal initially. It is apparent from the result that decreasing trend in plasma LH found, and the level was significantly (p<0.05) lowest on day 135 of gestation. Juarez *et al*.[20] reported a higher concentration of LH in mid-pregnancy and the lowest value at the end of gestation in goats but in our finding the concentration of LH started declining from day of estrus up to 135 day of gestation and again increased on expected day of parturition and 2 day after parturition. Our results agree with the findings of Namita [22]. ANOVA revealed that there was a significant difference between early, mid, and late gestation (p<0.05). The initial decrease in the concentration of LH from day of estrus up to day 135 may be due to the high rise of progesterone for maintaining pregnancy whether on the expected day of parturition there is an increase in the concentration of estrogen which enhances the production of LH high feedback mechanism.

The average of total plasma FSH concentration in different treatment groups recorded at different intervals is presented in Table-2. ANOVA revealed a non-significant variation observed on plasma FSH concentration among all the groups. However, it decreased from the day of estrus until 2 days after parturition in all the groups. Our results are in agreement with the findings of Xia *et al*. [23] in sheep. As FSH enhanced the development of medium-sized follicles in the goat by suppressing the apoptosis of granules cells via increasing production of insulin-like growth factor-1 (IGF-1) and steroids, possible through the PKA pathway [24]. As there is decreased in the level of FSH in our experiment shows that inhibin is a key hormone in the regulation of follicular development through regulation of endogenous FSH secretion during early pregnancy in goats [25]. ANOVA revealed that there was a significant difference between early and late gestation (p<0.05). The difference between early and mid was not significant but mid and late pregnancy differed significantly (p<0.05). It might be possible that the level of inhibin is high during pregnancy that may cause the low production of FSH during the gestation period.

The initial concentration of plasma triiodothyronine in different treatment groups recorded at the different interval is presented in Table-3 (1.13±0.02, 1.23±0.47, 1.17±0.21 ng/ml). The T_3_ concentration did not show any significant variation within and between the groups, although there was a decreasing trend in all the groups. It was significantly decreased (p<0.01) from day 1 to day 135 of gestation and again increased on expected day of parturition and 2 days after parturition. The level of plasma triiodothyronine increased significantly on expected day of parturition in all groups. It was observed that the plasma triiodothyronine concentration varied significantly within all the three groups different period of observation. Our findings agree with the findings of Patil [26] in pregnant goats and Ludri and Sharma[27] in Alpine X Beetal cross bred goats. Mabjeesh *et al*.[28] reported in Sagneu goats concentration triiodothyronine in plasma were greater during lactation in the short-day treatment. They concluded this maybe due to increased secretion of IGF-1.

**Table-3:** Mean±SE of plasma triiodothyronine (T3), thyroxine (T4), cortisol of black Bengal goats in different groups at different periods.

Parameters	Experimental group	Period of observations						

		Day 1 estrus	Day 20	Day 45	Day 90	Day 135	Expected day of parturition	2 days after parturition
Plasma triiodothyronine (T3) (ng/ml)	Group-I	1.13±0.02	1.19±0.24	1.04±0.10	1.02±0.52	1.11±0.62	1.45±0.71	1.78±0.49
	Group-II	1.23±0.47	1.23±0.48	1.15±0.30	1.13±0.45	1.27±0.40	1.38±0.52	1.68±0.59
	Group-III	1.17±0.21	1.13±0.33	1.20±0.32	1.33±0.33	1.02±0.11	1.43±0.45	1.72±0.53
Thyroxine (T4) (ng/ml)	Group-I	18.64±0.72^a^	18.60±1.10^a^	19.54±1.50^a^	21.42±1.26^ab^	21.30±1.09^ab^	24.50±2.06^b^	20.34±1.28^ab^
	Group-II	20.80±2.03	21.73±2.62	22.01±1.17	22.74±0.96	20.93±2.13	23.70±1.79	21.56±1.13
	Group-III	17.68±1.11	18.39±0.99	19.22±1.61	19.00±1.62	18.95±1.61	20.09±2.06	18.66±1.06
Cortisol (ng/ml)	Group-I	4.00±0.83^a^	5.12±0.95^a^	10.25±0.71^ab^	14.26±1.51^b^	21.50±2.69^c^	25.44±4.50^c^	3.75±1.89^a^
	Group-II	3.58±0.25	4.48±0.73^a^	9.64±1.64^b^	12.37±1.81^b^	23.11±1.25^c^	25.49±2.21^c^	3.59±1.23^a^
	Group-III	3.88±1.28	5.04±1.20	10.88±2.73^b^	11.84±1.25^b^	20.61±1.72^c^	24.68±2.04^c^	3.18±0.95^a^

Mean values bearing different superscripts in a row differ significantly (p<0.05), SE=Standard error

The average of total plasma thyroxine concentration in different treatment groups recorded at different interval is presented in Table-3. ANOVA revealed a significant variation (p<0.05) in plasma thyroxine concentration on day 1 and expected the date of parturition. The plasma level of thyroxine significantly (p<0.01) increases in all the groups during all periods of observation. Our findings agree with the finding of Alwan *et al*. [29] who reported inthe ewe. Our results are in range as reported by Patil [26] and Kaneko *et al*. [30]. Our observations also agree with the finding of Nazifi *et al*. [31] studied on thermal stress on serum biochemical and their correlation with thyroxine (T_4_) on healthy Iranian fat-tailed sheep. They reported the concentration of thyroxine in cold condition was higher than heat stress.

The average of total plasma cortisol concentration in different treatment group recorded at different intervals is presented in Table-3. ANOVA revealed a significant (p<0.05) variation in plasma cortisol concentration in all the groups increased significantly until day of parturition and dropped significantly (p<0.01) on 2 days after parturition in all the groups. Increase in plasma cortisol level on day 135 of gestation was reported by several workers [31,32] investigated the effect of thermal stress on cortisol concentration in Iranian fat-tailed sheep. There were no significant differences in the concentration of cortisol at either heat stress or cold stress. Our findings do not agree with the report of Nazifi *et al*. [31]. Sireli *et al*. [33] reported the level of cortisol increase up to the 5^th^ month of pregnancy and decreased after parturition similar to our findings.

## Conclusion

From the present study, it can be concluded that rise intemperature did not have any significant effect on metabolic and reproductive hormonal concentration during different stages of gestation in black Bengal goat.

## Authors’ Contributions

BK and AKI have conceived, planned and designed the study and conducted the research. BK, AKI, and PKC analyzed and kept a due record of the data. Manuscript was framed and drafted by BK, AKI, PKC, and TA. All authors read and approved the final manuscript.
